# Assessment of surgery delay-associated risk in resectable stages I–IIIA non-small-cell lung cancer

**DOI:** 10.1097/JS9.0000000000001612

**Published:** 2024-05-09

**Authors:** Zhenyi Niu, Yijun Wu, Runsen Jin, Hecheng Li

**Affiliations:** a Department of Thoracic Surgery, Ruijin Hospital, Shanghai Jiao Tong University School of Medicine, Shanghai; b Division of Thoracic Tumor Multimodality Treatment, Cancer Center, West China Hospital, Sichuan University; c Laboratory of Clinical Cell Therapy, West China Hospital, Sichuan University, Chengdu, Sichuan, People’s Republic of China

*Dear Editor,*

As the clinical practice published in *International Journal of Surgery*, surgical treatment delay occurs commonly worldwide because of limited medical resources, which has been further exacerbated by the lasting COVID-19 pandemic^1,2^. Various types of scheduled surgeries may have experienced delays, especially surgeries for early-stage tumors. Theoretically, timely interventions for resectable non-small-cell lung cancer (NSCLC) patients may maximize therapeutic potentials for decreasing recurrence and improving prognosis. Despite several studies that demonstrated some associations between delayed surgery and NSCLC patients’ outcomes^3–5^, whether the short-term delay has a significant effect and how long it is supposed to be for safety remains unclear. Thus, the objective of this study was to assess surgery delay-associated impacts on NSCLC outcomes using the largest clinical cohort to date.

Here, we reported a population-based cohort of 50 522 adults receiving standard pulmonary resection for clinical stages I–IIIA NSCLC that were diagnosed from the Surveillance, Epidemiology, and End Results Program database between January 2004 and November 2019. Surgery delay was defined as the time interval between the diagnosis date and the surgery date, which was recorded by month. For example, 0 month represented 0–29 days, and 1 month represented 30–59 days, and so on. Exclusion criteria were patients with surgery delay longer than 12 months, receiving chemotherapy or radiotherapy and undergoing more complex surgical resection.

The median age of the 50 522 enrolled patients at diagnosis was 69 (interquartile range: 62–75) years with an average surgery delay of 1.24 months (Table 1). Whites (84.5%), adenocarcinomas (62.5%), and stage I cases (84.8%) accounted for the highest proportions. Most patients underwent lobectomy (80.1%), followed by wedge resection (15.1%) and segmentectomy (4.8%). The surgery delay time of more than 80% of cases was within 3 months, with 18 094 (35.8%) patients shorter than 30 days, 15 28 (30.7%) patients within 30–59 days, and 10 032 (19.9%) patients within 60–89 days.

**Table 1:** Clinical characteristics and surgery delay of NSCLC patients.

Characteristic	Patients, No. (%) (*N*=50 522)	Average surgery delay, months
Age, years	69 (62, 75)	1.24
Sex		
Male	22 413 (44.4)	1.28
Female	28 109 (55.6)	1.21
Race		
White	42 695 (84.5)	1.21
Black	3843 (7.6)	1.41
Others	3984 (7.9)	1.39
Histologic type		
ADC	31 579 (62.5)	1.22
SCC	11 529 (22.8)	1.30
Others	7414 (14.7)	1.20
Stage		
I	42 846 (84.8)	1.21
II	5672 (11.2)	1.38
IIIA	2004 (4.0)	1.42
Surgical resection		
Wedge	7638 (15.1)	1.10
Segmentectomy	2424 (4.8)	1.14
Lobectomy	40 460 (80.1)	1.27
Delay time, months (days)		
0 (1–29)	18 094 (35.8)	–
1 (30–59)	15 528 (30.7)	–
2 (60–89)	10 032 (19.9)	–
3 (90–119)	3934 (7.8)	–
4 (120–149)	1454 (2.9)	–
5 (150–179)	666 (1.3)	–
≥6 (≥180)	814 (1.6)	–

Values are presented as median (interquartile range) or *n* (%).

ADC, adenocarcinoma; NSCLC, non-small-cell lung cancer; SCC, squamous cell carcinoma.

The restricted cubic spine (RCS) curve demonstrated a non-linear association between the surgery delay and estimated survival (Fig. 1A). Both all-cause (Fig. 1B) and cancer-specific (Fig. 1C) mortality risks increased drastically and simultaneously within the delay range of 3 months (0–89 days), followed by a much slower increment after 90 days. Therefore, we propose classifying all patients into three groups based on death risk stratification of the surgery delay: low-risk (delay ≤29 days), intermediate-risk (30–59 days), and high-risk (≥60 days). Compared with low-risk group, all-cause and cancer-specific mortality risk increased 6.2% (95% CI: 3.0–9.5%) and 17.6% (95% CI: 10.3–25.5%) in the intermediate-risk group, and by 18.3% (95% CI: 14.8–21.9%) and 40.5% (95% CI: 32.1–49.5%) in the high-risk group, respectively (*P*<0.001; Fig. 1).

**Figure 1:**
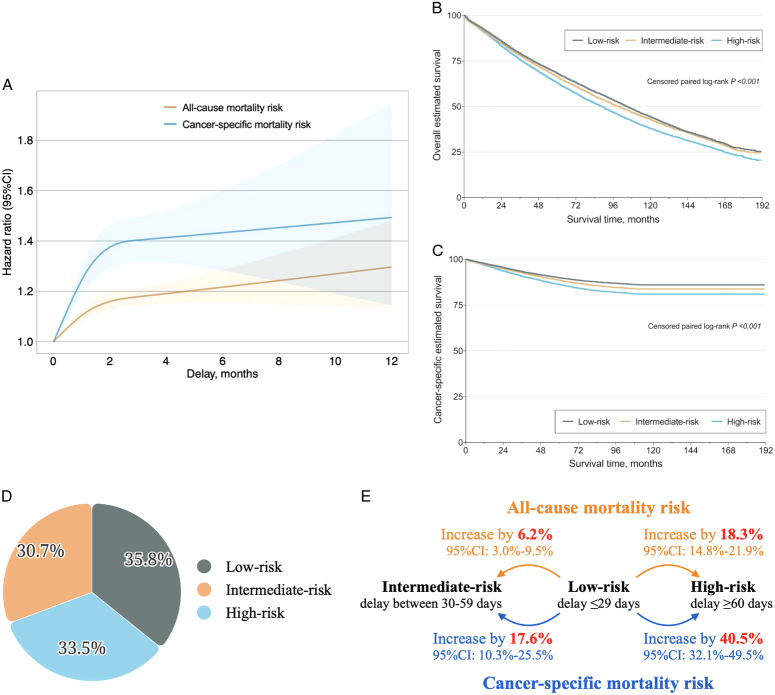
(A) Restricted cubic spline model presents the hazard ratio of surgery delay on estimated survival when compared with patients who received surgery within 29 days after diagnosis. The orange line indicates all-cause mortality risk, while the blue line indicates cancer-specific mortality risk. The 95% confidence interval of the hazard ratio is also illustrated. 0 month, 0–29 days; 1 month, 30–59 days; 2 months, 60–89 days, and so on. The overall estimated survival (B) and cancer-specific estimated survival (C) of patients with resectable stages I–IIIA non-small-cell lung cancer with low-risk (delay ≤29 days) vs. intermediate-risk (delay between 30 and 59 days) vs. high-risk (delay ≥60 days). (D) Percentage of patients in different risk groups. (E) All-cause and cancer-specific risks between the low-risk group and the intermediate-risk/high-risk groups.

Using the largest cohort to date, this population-based study confirmed the associations of the surgery delay with unfavorable outcomes in patients with resectable NSCLC. This study was the first to identify that surgery delay could significantly increase all-cause and cancer-specific mortality risk even beyond only 1 month (≥30 days), which may overcome limitations of previous studies attributed to small sample size and large delay cutoff^3–5^.

We observed that approximately only one-third of patients could receive surgical treatment within the first month after diagnosis, suggesting that most of them were exposed to increased surgery delay-associated risk. In the context of favorable prognosis among early-stage resectable NSCLC patients, it is highly recommended to minimize treatment delay by improving multifaced interventions to achieve comparable outcomes, such as the settlement of socioeconomic limitations, optimization of the diagnosis process, reasonable allocation of healthcare resources, and so on.

Surprisingly, the surgery delay-related mortality risk increased the fastest within a 3-month delay, indicating that there might be disease progression during the period in some cases that the most vulnerable to the increased risk. However, when ranging from 4 to 12 months, the risk increased more slowly. Therefore, these results further suggested the necessity for patients to receive surgical treatment within the safest period of 1 month. Moreover, we also observed synchronous changes of all-cause and cancer-specific mortality risk as the delay time increased, which might indicate a closer relationship between surgery delay and cancer-specific events instead of non-cancer ones. Overall, our findings firstly reveal that controlling the surgery delay within 1 month is essential for resectable NSCLC to avoid worse outcomes.

## Ethical approval

Not applicable.

## Consent

Not applicable.

## Sources of funding

This study was supported by the Postdoctoral Fellowship Program of CPSF (GZB20230481), National Natural Science Foundation of China (82072557, 81871882, 82303773), National Key Research and Development Program of China (2021YFC2500900), Shanghai Municipal Commission of Health and Family Planning Outstanding Academic Leaders Training Program (2017BR055), Shanghai Municipal Education Commission-Gaofeng Clinical Medicine Grant (20172005) and Natural Science Foundation of Shanghai (22ZR1439200).

## Author contribution

Y.W. and Z.N.: wrote the paper; R.J. and Z.N. analyzed the data; H.L.: study concept and review and editing.

## Conflicts of interest disclosure

There are no conflicts of interest.

## Research registration unique identifying number (UIN)

Not applicable.

## Guarantor

Professor Hecheng Li.

## Data availability statement

All data generated from this study have been included in the manuscript.

## Provenance and peer review

Not applicable.
